# Time after time: the chronology of photosynthesis inhibition by ethylene

**DOI:** 10.1093/plphys/kiae033

**Published:** 2024-01-21

**Authors:** Marieke Dubois

**Affiliations:** Assistant Features Editor, Plant Physiology, American Society of Plant Biologists; Department of Plant Biotechnology and Bioinformatics, Ghent University, 9000 Ghent, Belgium; VIB Center for Plant Systems Biology, 9000 Ghent, Belgium

Just like humans, plants use hormones as systemic signaling molecules to coordinate development and responses to the environment. The phytohormone ethylene is a small volatile molecule that is referred to as an aging hormone because of its key role in climacteric fruit ripening, leaf senescence, and abscission. In addition, ethylene synthesis is induced in plants experiencing biotic or abiotic stress, and ethylene plays a pivotal role in reducing growth and photosynthesis under such adverse conditions. Regulating photosynthesis is complex, as photosynthesis is the outcome of a fine-tuned choreography of multiple cellular and molecular factors: light capture enabled by leaf and chloroplast movements, gas exchange coordinated by stomatal closure, chlorophyll content in mesophyll cells, regulated activity of the photosystem machinery, and sugar partitioning (Wang et al. 2013). Previous studies have shown that ethylene affects several, if not all, of these factors important for photosynthesis and does so in a species-dependent manner (Ceusters and Van De Poel 2018). However, the precise mode of action and chronology by which ethylene inhibits photosynthesis remains largely underexplored.

In the current issue of *Plant Physiology*, Mohorović et al. (2023) elegantly combined physiological and molecular analyses to construct a timeline of ethylene-mediated effects on photosynthesis in tomato leaves. To monitor photosynthesis in real time upon ethylene treatment, tomato plants were grown in airtight Plexiglass boxes equipped with a CO_2_ sensor and connected to an ethylene supply system (Fig. 1A). After 8 hours to 4 days of ethylene treatment, leaf movements, stomatal conductance, ethylene signaling, sugar concentrations, CO_2_ content, and other parameters were scored. Subsequent data integration demonstrated that inhibition of photosynthesis by ethylene occurred in three major temporal steps (Mohorović et al. 2023).

**Figure 1.**
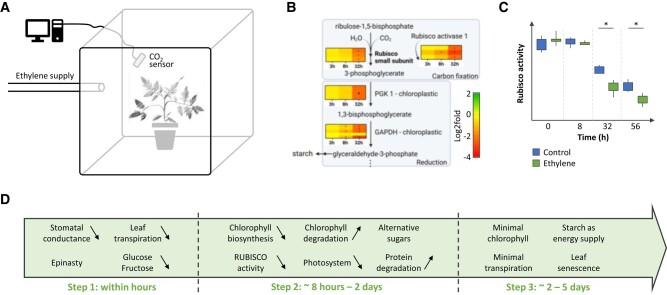
Inhibitory effect of ethylene on photosynthesis in tomato leaves. **A)** Schematic representation of the experimental setup used in Mohorović et al. 2023 to measure photosynthetic changes upon ethylene treatment. Young tomato plants were grown in Plexiglass boxes equipped with a tube for ethylene supply and a CO_2_ sensor for real-time measurement of the photosynthetic activity. **B)** Example of the RNA sequencing analysis performed by Mohorović and colleagues, mapping the expression of genes related to photosynthesis. Here, the downregulation of the Rubisco subunits and activase genes as well as of several genes involved in carbon fixation is shown. **C)** Rubisco activity measurements were combined with transcriptome analysis, showing significantly reduced (*) Rubisco activity after 32 h of ethylene treatment. **D)** Timeline for the ethylene-mediated photosynthesis inhibition, which was found to occur in three main steps. For details about the affected processes, see main text. Data in B–C adapted from Mohorović et al. 2023.

The initial responses were detected in the transcriptome, by performing RNA sequencing on a time series of young leaves after ethylene treatment. Among the earliest changes detected (3 to 8 h of ethylene treatment), multiple genes encoding the formation or activation of the rubisco complex were downregulated (Fig. 1B). To confirm these molecular findings at the physiological and phenotypic levels, Mohorović and colleagues measured changes in the leaf's physiology using a stomatal conductance porometer clipped onto the young, photosynthetically active leaf and observed ethylene quickly reduced stomatal conductance. This reduced leaf transpiration continued beyond 8 h. Next, the authors measured leaf movements upon ethylene treatment, as light capture can be decreased by the downward bending of leaves, a process called epinasty. Upon 8 h of ethylene treatment, the tomato leaves showed epinastic bending, reducing the canopy cover. Finally, by measuring the levels of soluble sugars in leaves, a reduction in both glucose and fructose concentrations was observed, likely resulting from the decreased transpiration.

The second response phase of ethylene-mediated photosynthesis inhibition occurred between 8 h and 2 days of ethylene treatment. Some of the earlier observed effects were further amplified: the leaf transpiration further decreased, and the canopy cover reached its minimum. The downregulation of Rubisco formation and activation genes observed earlier led to a reduction of Rubisco activity after 32 h of ethylene treatment (Fig. 1C). Molecularly, more genes were differentially expressed, with approximately 20% of the tomato transcripts altered by ethylene treatment. Possibly as a secondary effect of the reduced light capture resulting from epinasty, chlorophyll biosynthesis genes were downregulated and chlorophyll degradation genes were upregulated. Electron transport chain, photosystem, and light-harvesting complex genes, key for the light reactions of photosynthesis, were also further downregulated. Despite the reduced carbon fixation, sucrose levels remained constant, which could be attributed to increased sucrose import from older leaves. Finally, because all developmental and metabolic changes induced by ethylene require energy, and energy is limited by reduced photosynthesis, alternative hexoses could be metabolized and proteasome genes were upregulated in order to degrade proteins into amino acids, some of which could be used as energy supply.

Finally, for the longer term (2 to 8 days), ethylene dramatically reduced both leaf transpiration rate and chlorophyll content, further dampening photosynthesis in young leaves. Reduced photosynthesis resulted in lower starch reserves: plants used more starch as energy supply, which was detected in the transcriptome data by the upregulation of starch catabolism genes paired with the downregulation of starch biosynthesis genes. Ultimately, senescence was prematurely initiated in the young leaves as shown by expression of senescence-associated genes.

Altogether, the work performed by Mohorović and colleagues revealed the chronology of ethylene-mediated photosynthesis inhibition in young tomato leaves. Their approach defined a 3-step response consisting of: (1) a reduction in the capture of CO_2_ and light, resulting in a rapid decline of soluble sugars; (2) a transcriptional downregulation of genes related to photosynthesis, coupled to the upregulation of genes necessary to gather energy from other sources, perhaps sucrose or proteins; and (3) a long-term starch and chlorophyll breakdown resulting in leaf senescence (Fig. 1D). Adding a time factor to the extensively studied ethylene response is crucial, as hormones often affect temporary or dynamic processes (Waadt et al. 2022) that can only be detected by time-course experiments, as was elegantly performed in Mohorović et al. 2023. This work paves the way toward further molecular characterization of the regulatory cascades linking the different steps of the ethylene-mediated inhibition of photosynthesis.

## Dive Curated Terms

The following phenotypic, genotypic, and functional terms are of significance to the work described in this paper:

electron CHEBI: CHEBI:10545
